# Drastic drop in blood flow of extracorporeal membrane oxygenation induced by an excessive increase in intra‐abdominal pressure in an obese patient

**DOI:** 10.1002/ccr3.2405

**Published:** 2019-08-30

**Authors:** Kenji Kandori, Hiromichi Narumiya, Ryoji Iiduka

**Affiliations:** ^1^ Japanese Red Cross Society Kyoto Daini Hospital Kyoto Japan

**Keywords:** extracorporeal membrane oxygenation, intra‐abdominal pressure, obesity, supine hypotensive syndrome

## Abstract

Obesity and conditions that increase intra‐abdominal pressure (IAP) should be considered as risk factors for reduced extracorporeal membrane oxygenation (ECMO) blood flow (BF) drastically. For obese patients on ECMO, effective IAP control and risk factor assessment is necessary to prevent excessive IAP elevation and subsequent drop in ECMO BF.

## 1 INTRODUCTION

Several studies exploring the relationship between obesity and extracorporeal membrane oxygenation (ECMO) efficacy and outcomes suggest that obesity is not a risk factor for in‐hospital mortality of patients requiring support with veno‐venous (VV)‐ECMO; thus, obesity is proposed not be considered as a contraindication to VV‐ECMO initiation.^1^, ^2^, ^3^, ^4^ However, ECMO management in obese patients poses special challenges, such as cannulation techniques and choice of the appropriate cannula size.^4^ We herein report the first case of a sudden drop in ECMO blood flow (BF) due to an abrupt increase in intra‐abdominal pressure (IAP) of an obese patient. We also discuss the underlying mechanism.

## 2 CASE REPORT

A 55‐year‐old male with dyspnea who was diagnosed with severe pneumonia was transferred to our hospital. The patient's height was 176 cm and weight was 120 kg, with a body mass index (BMI) of 38.7. The computed tomography at the previous hospital showed bilateral pneumonia, which was determined to be worsening by X‐ray at our hospital. Arterial blood gas analysis on 15 L/min O_2_ using a bag valve mask was as follows: pH, 7.28; PaCO_2_, 64.7 mm Hg; and PaO_2_, 44.7 mm Hg. His hypoxia did not improve after intubation, VV‐ECMO was initiated. Percutaneous cannulation was performed using a 23‐French drainage cannula for access via the right femoral vein and a 19‐French cannula for return via the right internal jugular vein without complications. ECMO was initiated at a BF of 5.0 L/min and a sweep gas flow of 5.0 L/min in 100% O_2_ per oxygenator. The lungs were rested with pressure‐controlled ventilation during ECMO, permitting support with a tidal volume of 100 mL. The patient was under sedation, rocuronium was used for muscular relaxation, and meropenem was used for antibiotic therapy. His hemodynamics was stable without catecholamine support.

On day 6, the patient had not defecated since admission, and his weight was 132 kg, and the rocuronium was discontinued. Several hours later, ECMO BF, which was maintained at approximately 3.0 L/min with a rotational speed of 2560 rpm, dropped abruptly to 0.5 L/min. Increasing the rotational speed to 3000‐3500 rpm did not recover ECMO BF. Potential displacement of the ECMO catheter was ruled out by the absence of any abnormalities by echography. First, we reduced the rotational speed to 500 rpm and then increased to 2800 rpm, the ECMO BF was recovered and stable similar to that prior to this episode. According to the review of this episode and abdominal X‐ray, the reason of the sudden drop of ECMO BF was that the patient had strained suddenly and strongly during defecation. After addressing the bloating and establishing a defecation routine, the patient did not experience another episode of a drop in ECMO BF.

## 3 DISCUSSION

The current case illustrates three important clinical issues. First, in obese patient, a high IAP decreases ECMO BF like supine hypotensive syndrome. Second, in that condition, an excessive increase in IAP can lead to defective drainage and drastic drop in ECMO BF. Third, effective IAP control and risk factor assessment is necessary to prevent the phenomenon.

Supine hypotensive syndrome can occur in pregnant women or patients with abdominal masses who lie on their back due to the compression of the uterus or the mass, respectively, on the inferior vena cava (IVC), leading to decreased blood return to the right atrium and decreased cardiac output. In the current case, an excessive abdominal distension and increasing in IAP led to the compression of IVC. He had not defecated since the hospital admission and his abdominal cavity content became larger. The large abdominal cavity content and his extensive abdominal fat were likely to have led to the excessive abdominal distension and the increasing in IAP. BMI is positively correlated with IAP.^5^, ^6^, ^7^ A possible explanation for higher pressures in the obese is that there could be a direct mass effect from the intra‐abdominal adipose tissue itself on the measurement of IAP.^8^ Prior to the first sudden drop in ECMO BF, the BF had decreased slightly when the patient was in the right lateral position, which had improved when he switched to the left lateral position, suggesting that he was potentially in condition of supine hypotensive syndrome due to the excessive abdominal distension.

**Figure 1:**
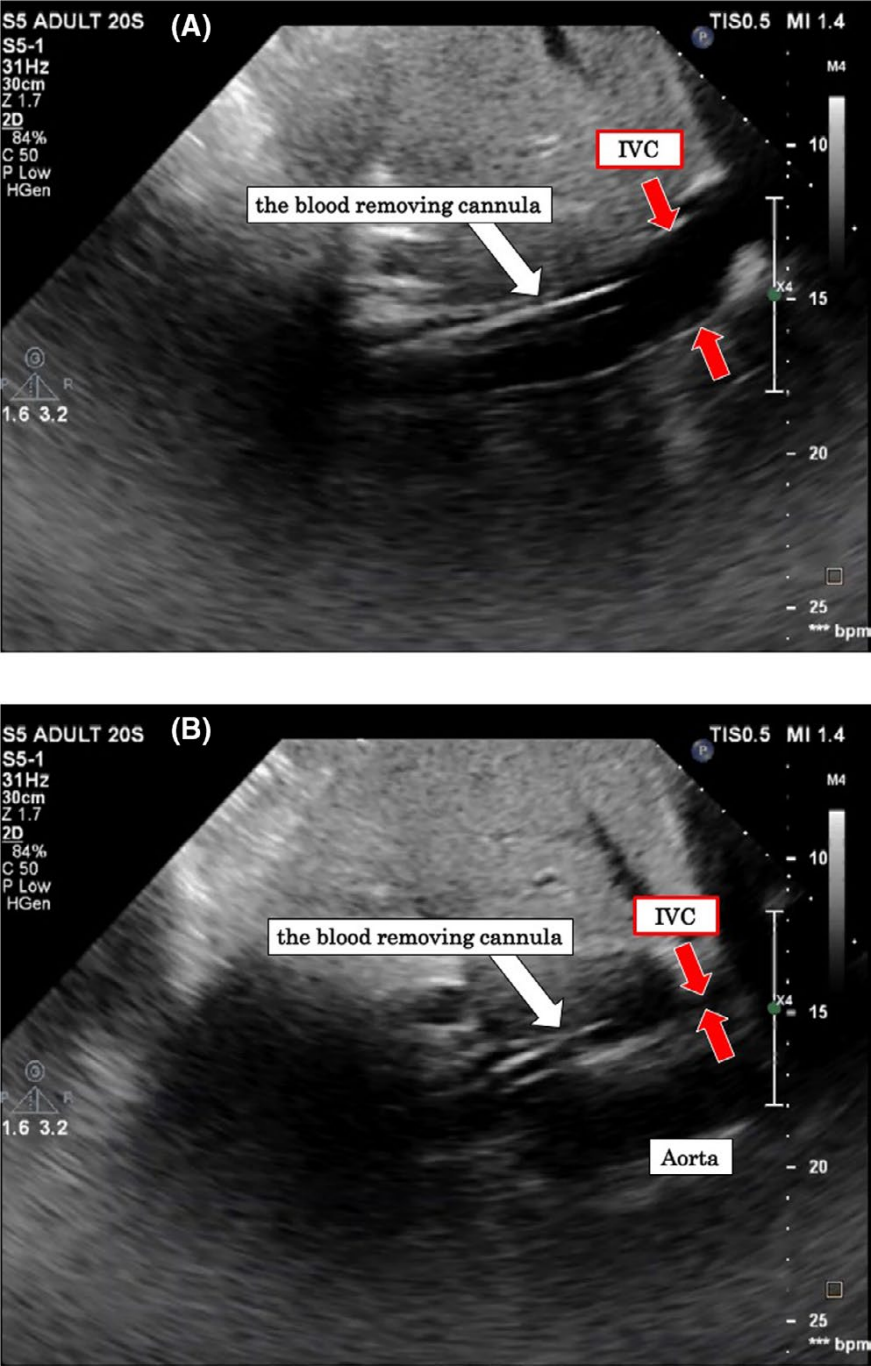
Ultrasonographic findings during reproduction of the episode. The diameter of the inferior vena cava was maintained, and no collapse was observed under baseline conditions (A). The inferior vena cava collapsed following straining (B). At the time of straining, the drainage of blood via the blood‐drainage cannula was interrupted, and a drop in blood flow of extracorporeal membrane oxygenation was induced

Second, in the condition like supine hypotensive syndrome, an excessive increase in IAP can lead to defective drainage and drastic drop in ECMO BF. The discontinuation of rocuronium led to the contraction ability of the abdominal wall and a gain in muscle strength, which together led to an increase in IAP. He was powerful, and the strength of straining was also great, and this led to an extreme increase in IAP. The excessive abdominal distension and the extreme increase in IAP compressed and obstructed IVC, which reduced the blood return. Consequently, this disrupted the blood drainage via the blood‐drainage cannula, the tip of which was placed at the IVC. To confirm this phenomenon, we asked him to flex his abdominal muscles while holding his breath, which led to a drop in ECMO BF due to an increase in IAP. The collapse of the IVC due to straining was confirmed by ultrasonography (Figure 1).

Third, a drop in ECMO BF should be considered as a possibility in obese patients, and IAP control is important as a precaution. Conditions that cause an increase in IAP should be considered as risk factors for a drop in ECMO BF. Preventative measures, such as muscle relaxant and defecation habits, and IAP monitoring should be considered in at‐risk patients.

The possibility of the tip of the blood‐drainage cannula adhering to the vessel wall should be considered if ECMO BF is not improved by release after the strain; to address this possibility, the rotational speed should be reduced once, followed by an increase, for an attempt to improve ECMO BF. The first drop in BF by straining during defecation of the patient did not improve spontaneously. It is possible that, although the rotational speed was increased to restore BF, this maneuver led to the adherence of the blood‐drainage cannula tip to the IVC wall.

## 4 CONCLUSION

This case of an obese patient receiving ECMO treatment who experienced a sudden drop in ECMO BF following an abrupt increase in IAP highlights the significance of risk assessment and adequate IAP control for the prevention of this phenomenon.

## CONFLICTS OF INTEREST

There is no conflict of interest.

## AUTHOR CONTRIBUTIONS

Dr Kenji Kandori: served as the major decision‐maker during the treatment and wrote the paper. Dr Hiromichi Narumiya: served as the major decision‐maker during the treatment. Dr Ryoji Iiduka: served as the major decision‐maker during the treatment. Kenji Kandori: served as the guarantor. All authors have read and approved the final manuscript.

## ETHICAL APPROVAL OF THE RESEARCH PROTOCOL

Ethical approval was exempted for case reports by our institution.

## INFORMED CONSENT

Written informed consent was obtained from the patient for publication of this case report and any accompanying images.
